# Oxygen Saturation Target Achievement in Preterm Infants <32 Weeks’ Gestation with 9^th^ Edition NRP Guidelines: A Multicenter Observational Study in Latin America

**DOI:** 10.21203/rs.3.rs-9487761/v1

**Published:** 2026-05-21

**Authors:** Maria Favareto, Susana Rodriguez, Ignacio Sosa-Boye, Marcelo Cardetti, Augusto Sola, Satyan Lakshminrusimha, SMARTO2 Study Group

**Affiliations:** Ibero American Society of Neonatology; UC Davis; Ibero American Society of Neonatology

## Abstract

**Objective:**

To evaluate delivery room oxygen practices based on the 9^th^ edition of Neonatal Resuscitation Program and preductal SpO_2_ at 5 minutes in preterm infants <32 weeks’ gestation

**Study Design:**

Prospective observational study in 9 centers across 6 Latin-American countries. Data included initial FiO_2_, changes during stabilization, and SpO_2_ at 5 minutes.

**Results:**

Among 173 infants, initial FiO_2_ was 0.3 in 71% (range:0.3–1.0). At 5 minutes, 18% were within target (SpO_2_ 80–85%). SpO_2_ was <80% in 16%, between 86–95% in 49% of infants and >95% in 17%. FiO_2_ was increased in 55% and 35% of infants received ≥0.6 FiO_2_ by 5 minutes. Among 41 infants ≤26 weeks, initial FiO_2_ varied (0.3 –71%, 0.4–0.5–12%, 0.6–0.9–15% and 1.0–2%) and was titrated up in 73%.

**Conclusion:**

With adaptation of initial FiO_2_ range suggested by 9^th^ edition NRP (0.3–1.0), two-thirds of preterm infants exceeded recommended SpO_2_ targets, suggesting potential hyperoxia exposure.

## Introduction

Oxygen administration during neonatal resuscitation requires balancing the risks of hypoxia and hyperoxia.^1^ International resuscitation guidelines have recently changed and currently recommend initiating resuscitation with ≥ 0.3 FiO_2_ and titrating based on pulse oximetry.^2^ Target SpO_2_ ranges during the first minutes after birth are derived from observational studies of healthy infants and are intended to guide safe oxygen delivery.^3^ Preterm infants, especially ≤ 28 weeks gestation are at high risk of hypoxemia (SpO_2_ <80%) by 5 minutes after birth.^4 5^

However, clinical practice often deviates from these recommendations. With the wide range of 0.3 to 1.0 FiO_2_ suggested by the Neonatal Resuscitation Program (NRP)^6^ for preterm infants < 32 weeks gestation, variability in initial FiO_2_ selection and titration may result in suboptimal oxygen exposure. Excess oxygen can contribute to oxidative injury, while insufficient oxygenation may impair transition.^7^

Several studies have demonstrated that not achieving an SpO_2_ of 80% by 5 minutes after birth increases the risk of mortality and morbidity.^5^ A recent study from California, USA suggested that nearly 70% of infants ≤ 23 weeks gestation at birth, ~ 50% of infants 24–26 weeks and ~ 40% 27–28 weeks did not achieve > 80% SpO_2_ by 5 minutes after birth.^4^

Data on real-world oxygen use practices, particularly in Latin America, are limited. This study evaluated oxygen use and target SpO_2_ achievement in preterm infants < 32 weeks’ gestation following the release of the 2025 guidelines suggesting the use of 0.3–1.0 FiO_2_ for initial resuscitation.^2, 6, 8^

## Methods

### Study Design and Setting

In late October 2025, an in-depth discussion of the new ILCOR suggestion for initial FiO2 during neonatal resuscitation was conducted at the Ibero American Society of Neonatology (SIBEN) congress in Cali, Colombia. Following this discussion, a prospective observational study was conducted in 9 institutions across 6 Latin American countries (Argentina, Bolivia, Colombia, Dominican Republic, Mexico and Peru). The study was initiated after the release and dissemination of 2025 resuscitation guidelines by the American Heart Association^8^ and American Academy of Pediatrics in October 2025.^2^ We focused on preterm infants < 32 weeks’ gestation requiring delivery room resuscitation or stabilization during the months of December 2025 to February 2026.

The study was exempt from the Institutional Review Board (IRB). No consent was obtained from parents as neonatal providers resuscitated infants as per current standard of care and de- identified data was collected as part of a quality improvement project.

### Data Collection

Collected variables included Initial FiO_2_, FiO_2_ adjustments, SpO_2_ at 5 minutes and simultaneous recorded FiO_2_ by gestational age subgroup. The primary outcome was percentage of infants within the preductal SpO_2_ 80–85% at 5 minutes. Secondary outcomes included distribution of SpO_2_, initial FiO_2_ practices by gestation and frequency of FiO_2_ adjustments by gestational age.

### Statistical Analysis

Descriptive statistics were used. Data are presented as counts and percentages.

## Results

**Figure 1:**
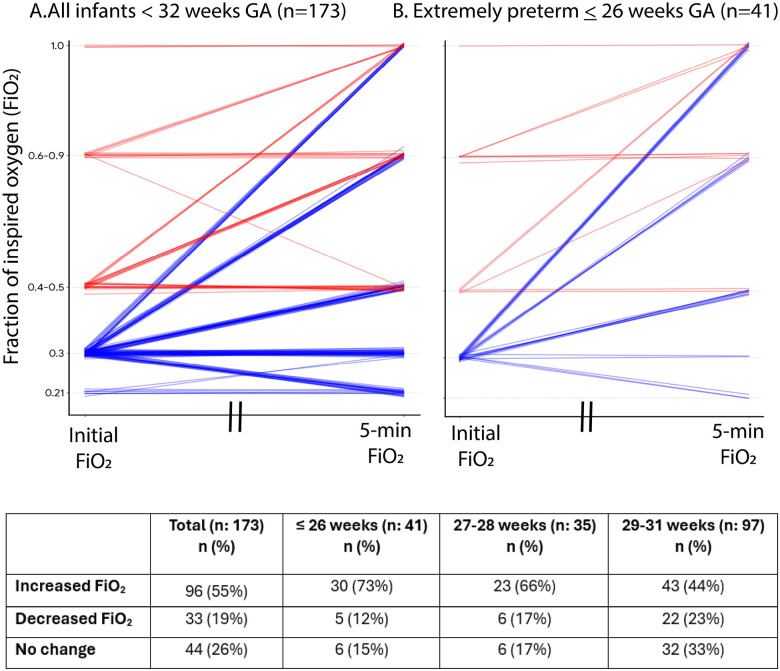
Initial FiO_2_ and FiO_2_ at 5 minutes among all preterm infants < 32 weeks gestation (A) and extremely preterm infants (≤ 26 weeks, B). The table in the inset shows directionality of change in FiO_2_ between birth and 5 minutes by gestational age categories

A total of 173 preterm infants < 32 weeks gestation were included; 41 of them were ≤ 26 weeks, 35 were 27–28 weeks and 97 were 29–31 weeks gestation. Initial FiO_2_ and range of SpO_2_ and FiO_2_ at 5 minutes are shown in Table 1A and as a spaghetti plot in Fig. 1A. Majority of infants received 0.3 as initial FiO_2_ (as recommended by the 8th edition of the Textbook of Neonatal Resuscitation).^9^ By 5 minutes, 60% of preterm infants received > 0.3 FiO_2_. Only 18% of infants achieved the desired target SpO_2_ of 80–85% by 5 minutes and 66% exceeded the desired target SpO_2_.

Among extremely preterm infants (< 26 weeks gestation), the results were similar although no infant received initial FiO_2_ of 0.21 at resuscitation and more infants needed escalation of FiO_2_ during the first 5 minutes after birth. More infants (34%) in the extremely preterm cohort required 1.0 FiO_2_ by 5 minutes compared to all infants < 32 weeks (14%, Fig. 1B and Table 1B).

**Figure 2:**
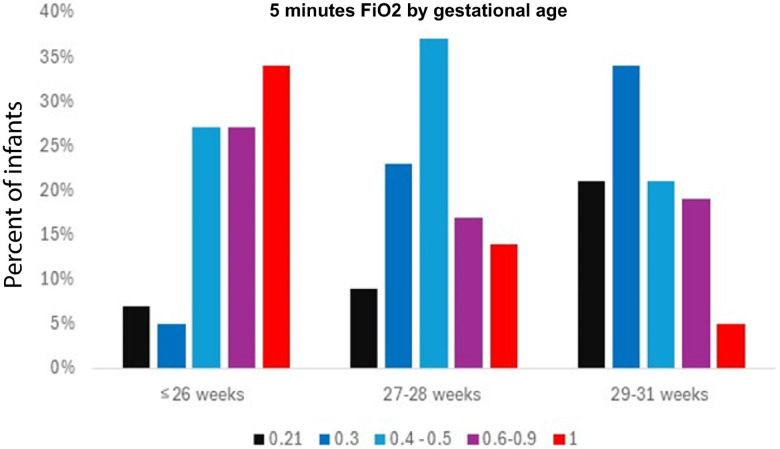
FiO_2_ Distribution at 5 minutes by gestational age categories

Inspired oxygen was titrated up in majority of infants, especially at lower gestational age (Fig. 1 inset). By 5 min, the most common FiO_2_ was 1.0 in the ≤ 26 weeks gestational age cohort, 0.4–0.5 at 27–28 weeks and 0.3 at 29–31 weeks (Fig. 2).

**Figure 3:**
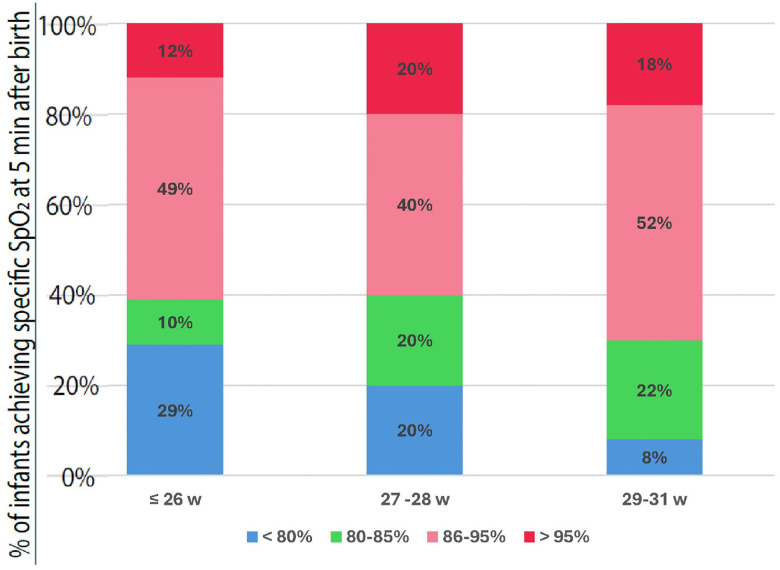
Percentage of infants achieving specific SpO_2_ ranges at 5 minutes after birth by gestational age categories

The SpO_2_ achieved at 5 minutes after birth is shown in Fig. 3. A small percentage of infants were at the desired target range (80–85%) and this percentage increased with advancing gestational age. The percentage of infants below the target range was 29% at ≤ 26 weeks gestational age cohort and decreased with advancing gestational age. At all gestational age groups, majority of infants exceeded 85% SpO_2_ at 5 minutes after birth (Fig. 3).

## Discussion

This study demonstrates that with the adaptation of revised NRP 9th edition guidelines^6^, there is a wide range of FiO_2_ utilized for initial resuscitation at birth. Although NRP 8th edition guideline-recommended initial FiO_2_ of 0.3 is widely adopted^9^, most infants exceed target SpO_2_ levels at 5 minutes. Only 18% achieved the target range, while two-thirds were above this range. These results on the frequency of SpO_2_ > 85% by 5 minutes are higher than those reported from California^4^ suggesting regional preferences and approach to titration of oxygen.

These findings align with prior studies showing difficulty in maintaining SpO_2_ within narrow target ranges during neonatal transition especially in extremely preterm infants ≤ 26 weeks gestation. The tendency toward higher oxygen saturation likely reflects clinician concern about hypoxia, leading to frequent upward titration of FiO_2_. The high rate of FiO_2_ escalation (55%) suggests that initial oxygen concentrations may be insufficient for some infants in the first couple of minutes after birth or that titration practices are overly aggressive. This was particularly evident in extremely preterm infants, where 73% received increased FiO_2_, and at 5 minutes of age only 10% were within target but 61% were above target. This may be associated with a delay in weaning FiO_2_ when SpO_2_ targets at 1–4 minutes are exceeded

Failure to achieve target SpO_2_ in ≤ 26 weeks infants highlight the challenges in this population. Their immature lungs and limited oxygen reserve may necessitate individualized strategies.

Importantly, the high proportion of infants exceeding SpO_2_ targets raises concern about hyperoxia exposure. Oxidative stress is implicated in neonatal morbidities including bronchopulmonary dysplasia, retinopathy of prematurity, and brain injury.^10, 11, 12, 13^ With the publication of the TORPIDO 30/60 trial showing no difference in outcome of death or morbidity between FiO_2_ of 0.3 vs. 0.6 to initiate resuscitation,^14^ there is a general tendency to use higher FiO_2_ of 0.9 to 1.0.^15^ This practice is supported by the individual patient metaanalysis (NETMOTION) showing a decrease in mortality with initiation of resuscitation with higher FiO_2_.^16^ Our study informs us to exercise caution with higher FiO_2_ for initiation of resuscitation in preterm infants and closely watch for the incidence of hyperoxia. This study also emphasizes the importance of appropriate titration (including weaning) during resuscitation.^17^

Our study has several limitations including lack of continuous SpO_2_ trends, absence of method used for O_2_ administration (i.e., airway pressure, interface) and of outcome correlations. We also did not standardize titration approaches to FiO_2_ in our centers. Finally, dissemination of the new NRP guidelines is not widespread in Latin America pending publication of the Spanish version of the Textbook of Neonatal Resuscitation at the time this study was conducted. Strengths of our study include multicenter design and real-world data with adoption of the revised NRP 9th edition suggestion for initial FiO_2_.

Future research in this field should focus on real-time oxygen titration algorithms and randomized trials evaluating the method and interfaces used for O_2_ administration. The use of advanced technology such as artificial intelligence-based algorithms and automated oxygen control systems should be evaluated. Finally, observational studies linking early oxygen exposure with outcomes from low-and-middle income countries are needed to evaluate the implications of initial FiO2 guidelines in these settings.

## Conclusion

Most infants < 32 weeks gestation exceeded recommended SpO_2_ targets at 5 minutes of age, irrespective of initial FiO_2_. This suggests potential hyperoxia exposure with the revised NRP guidelines and that quality improvement of FiO_2_ titration practices is needed.

## Supplementary Material

Tables are available in the Supplementary Files section.

This is a list of supplementary files associated with this preprint. Click to download.

- Table1AandB.docx

## Data Availability

Available upon request. SMARTO_2_ study group (in addition to the authors): Gonzalo Vega [Hospital Provincial de Rosario, Santa Fe - Argentina]; Maria Laura Alonzo [Clínica Universitaria Reina Fabiola, Cordoba-Argentina]; Taína Malena, Joselyn Acosta, Maria Castillo [Hospital Materno Dra Evangelina Rodriguez, Santo Domingo-Republica Dominicana]; Carmen Davila Aliaga, Elsa Torres Marcos, Instituto Nacional Materno Perinatal, Lima - Peru]; Javier Torres Muñoz, Clinica Versalles, Cali - Colombia]; Elizabet Fabbro y Sergio Conzolino [Maternidad Martin, Rosario, Santa Fe - Argentina]; Alberth Challapa Mancilla y Lili Ximena Aranibar Montoya [Hospital de la Mujer Dr. Percy Boland Rodriguez, Santa Cruz- Bolivia]; Monica Menzio, Gabriela Piguillem [Maternidad Provincial Dra Teresita Baigorria, San Luis Argentina], Regina Diaz Canejas y Jose Luis Ramirez Haua [Hospital Español, DF- Mexico]
